# The Association Between High Hemoglobin Levels and Pregnancy Complications, Gestational Diabetes and Hypertension, Among Palestinian Women

**DOI:** 10.7759/cureus.18840

**Published:** 2021-10-17

**Authors:** Haytham Abumohsen, Baraa Bustami, Abeer Almusleh, Osama Yasin, Ahmad Farhoud, Omar Safarini, Ammar Thabaleh, Mulham Sukhon, Zaher Nazzal, Basma Damiri

**Affiliations:** 1 Medicine, An-Najah National University, Nablus, PSE; 2 Community and Family Medicine, An-Najah National University, Nablus, PSE; 3 Faculty of Medicine and Health Sciences - Drug and Toxicology Division, An-Najah National University, Nablus, PSE

**Keywords:** maternal mortality, maternal morbidity, fasting blood sugar, high hemoglobin level, gestational hypertension, gestational diabetes

## Abstract

Introduction: Gestational diabetes mellitus (GDM) and hypertensive disorders of pregnancy (HDP) are the principal causes of maternal morbidity and mortality. The maternal morbidity and mortality burden for Palestinian women is relatively high, suggesting a substandard quality of care. Therefore, an early diagnosis of GDM and gestational hypertension (GH) can improve prenatal care for pregnant women and improve pregnancy outcomes. Previous studies demonstrated that elevated Hb levels in the first trimester indicate possible pregnancy complications and should not only be considered as good iron status. However, ethnic differences could play a role in determining the magnitude of the association. We hypothesized that high Hb levels (≥12.5 g/dl) in the first trimester (6-13 gestational weeks, GW) are associated with increased risk of fasting blood sugar (FBS) ≥126 mg/dl, systolic blood pressure (SBP) ≥140 mmHg, and diastolic blood pressure (DBP) ≥90 mmHg among pregnant Palestinian women visiting prenatal clinics in Palestine.

Methods: Medical records (N=5263) were reviewed for singleton pregnancies who had their first maternity care clinic visit (6-13 GW) at primary healthcare centers of the Palestinian Ministry of Health in the north of the West Bank in 2018 and 2019. Women were excluded if they had FBS ≥92 mg/dl, SBP ≥140 mmHg, DBP ≥90 mmHg, ultrasound-based gestational age >13 weeks, or who were previously diagnosed with diabetes mellites, GDM, hypertension, GH, taking drugs for these conditions, or were smoking during pregnancy. Hb levels in g/dl were divided to low (<11.0), normal (11-12.49), and high (≥12.5). The associations between high hemoglobin levels and pregnancy complications in pregnant women were assessed by calculating the odds ratios (OR) and their 95% confidence intervals (CIs) using logistic regression. P-values of <0.05 were considered significant.

Results: The final number of eligible records was 2565. Pregnant women with high Hb levels in the first trimester were at higher risk of high FBS (≥126 mg/dl; OR=2.99, 95%CI, [1.675-5.368]) and high systolic blood pressure (≥140 mmHg; OR=3.048, 95%CI, [1.252-7.421]) at 24 GW. Gravidity was significantly associated with decreased risk of high FBS (OR=0.838, 95%CI [0.704-0.991]).

Conclusion: Our findings suggest that Hb level at registration could be utilized in predicting the risk of GDM and HP among Palestinian women who never had a previous history of these conditions. The results of this study could have important clinical implications for early screening, which could improve preventive and curative health services to promote the health of pregnant women and children.

## Introduction

Gestational diabetes mellitus (GDM) and gestational hypertension (GH) significantly contribute to maternal, fetal, and neonatal morbidity and mortality [1]. The prevalence of GDM is rising worldwide and ranges from 1% to 20% [2]. During normal pregnancy, progressive insulin resistance develops during mid-pregnancy and progresses through the third trimester [3]. Globally, hypertensive disorders of pregnancy (HDP) are one of the leading causes of peripartum morbidity and mortality [4]. HDP complicates up to 2.73% of all pregnancies and is responsible for 10-15% of all U.S. maternal mortality [5]. It is associated with a spectrum of severity, ranging from mild pregnancy-induced hypertension to eclampsia [5]. Moreover, it is among the most significant and intriguing problems in obstetrics [5]. Palestinian women are at higher risk of pregnancy complications due to the stressful life they live [6,7]. The burden of maternal morbidity and mortality for Palestinian women is relatively high, suggesting a problem of substandard quality of care [6,7]. Moreover, HDP is under-treated among Palestinian women and is associated with an increased risk of cesarean section, preeclampsia (PE), antepartum hemorrhage, postpartum hemorrhage, and chronic hypertension [8]. Early diagnosis of GDM and GH can improve prenatal care for pregnant women during pregnancy and result in a satisfactory pregnancy outcome [1].

Hemoglobin (Hb) measurement is a routine standard test for evaluating physical status among pregnant women in their first visit to primary health care clinics [9]. Throughout normal pregnancy, blood volume expands by an average of 50% compared with the non-pregnant state [10]. This rapid expansion of blood volume starts in the first trimester of pregnancy [11]. Moreover, plasma volume increases more than the increase in red blood cell (RBC) mass, which produces a net decline in hemoglobin concentration during the first half of pregnancy. This is known as the physiologic anemia of pregnancy [11]. Hb concentration reaches the nadir in the second trimester of pregnancy because a concurrent increase does not match the increase in plasma volume in RBC mass increase [12]. Based on the World Health Organization (WHO) and the U.S. Centers for Disease Control and Prevention (CDC) guidelines, anemia in pregnancy has different cutoffs based on the trimester (first trimester: <11.0 g/dl; second trimester: <10.5 g/dl; and third trimester: <11 g/dl) [13] while normal values are assigned from 11 to <12.5 g/dl [14]. Physicians and health care providers give more attention to maternal anemia than high blood levels. Previous studies demonstrated that elevated Hb levels in the first trimester indicate possible pregnancy complications and should not be mistaken for good iron status [15-22]. They also indicated that Hb levels during early pregnancy play a role in predicting the risk of GDM and PE [16-19]. Studies investigated the association between high maternal Hb levels and adverse pregnancy outcomes are scarce and controversial, with no absolute cut-off values for high Hb levels [15-22]. The cutoffs used to define low or high hemoglobin concentrations in these studies differed considerably, which may have affected the likelihood of detecting relations with the outcomes [21]. Most often, only the most extreme cutoffs were significantly associated with pregnancy complications.

Further research is necessary to study and better understand the heterogeneity in the suggested cutoffs and risk factors associated with pregnancy outcomes. Moreover, it was suggested that ethnic differences could play a role in determining the magnitude of the association between high Hb and pregnancy complications [16]. Therefore, further investigation in different ethnicities was recommended. Based on the literature review, the assessment of high hemoglobin level by which cutoff should be taken as standard is still not clear. Taking into consideration the limitations of these studies, the existing literature is insufficient. The adverse effects of high Hb at registration among Palestinian pregnant women have not been previously investigated. Therefore, we conducted a retrospective study to investigate the association between maternal Hb levels in the first trimester (6-13 gestational weeks, GW) and adverse pregnancy outcomes (i.e., gestational hypertension and diabetes) among pregnant Palestinians. We hypothesized that high Hb levels (≥12.5 g/dl) in the first trimester (6-13 GW) are associated with an increased risk of fasting blood sugar (FBS) ≥126 mg/dl, systolic blood pressure (SBP) ≥140 mmHg, and diastolic blood pressure (DBP) ≥90 mmHg among pregnant Palestinian women visiting prenatal clinics in Palestine from January 2018 to December 2019. The results of this study could have important clinical implications for early screening, improving preventive and curative health services to promote healthy pregnant women. The abstract of this research was previously presented at the 11th International Palestinian Conference for Laboratory Medicine (IPCLM 11) on August 26, 2021.

## Materials and methods

**Figure 1:**
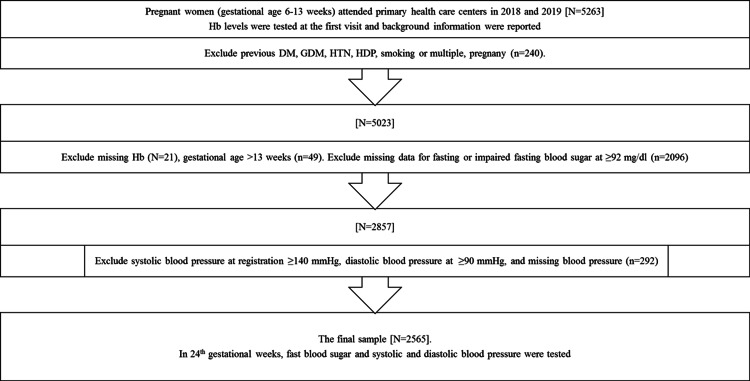
Flowchart of the participants Hb: hemoglobin, GW: gestational Week, FBS: fasting blood sugar, mg/dl: milligram per deciliter, g/dl: gram per deciliter, mmHg: millimeter of mercury, n or N: number

A cross-sectional study was performed in 2021 at primary healthcare centers of the Palestinian Ministry of Health (MoH) in Nablus, Jenin, Tulkarm, and Tubas; the largest four governorates out of six in the north of the West Bank/Palestine. Low Hb levels were defined according to WHO and CDC definition (Hb <11.0 g/dl) [13] while normal Hb was defined as Hb ranging between 11.0 and 12.49 g/dl and high Hb concentration ≥12.5 g/dl [14]. Based on WHO definition, fasting plasma glucose during pregnancy ≥92 to <126 mg/dl (gestational diabetes) or fasting plasma glucose ≥126 mg/dl (diabetes mellitus) [23]. Other high biochemical and medical levels were defined as the following: high systolic blood pressure ≥140 mmHg, and high diastolic blood pressure ≥90 mmHg. All medical records (N=5263) were reviewed for pregnant women who attended primary healthcare centers of the MoH in these governorates in the years 2018 and 2019. The year 2020 was excluded from the study due to the COVID-19 pandemic and quarantine. Out of 5263 records, 2698 medical records were excluded from this study as they met the exclusion criteria. Women were excluded if they had a history of a current or previous diabetes mellitus (DM), GDM, abnormal FBS, hypertension, GH, HDP, multi-pregnancies, and smoking during pregnancy. This includes women who had: FBS ≥92 mg/dl or missing values for FBS (N=2096), blood systolic blood pressure ≥140, diastolic blood pressure ≥90, or missing values for blood pressure (N=292), missing hemoglobin values (N=21), ultrasound-based gestational age more than 13 weeks (N=49), or who were previously diagnosed with DM/GDM/hypertension/gestational hypertension, or taking drugs for these conditions (N=240; Figure 1).

Statistical analyses were performed using IBM Corp. Released 2013. IBM SPSS Statistics for Windows, Version 22.0 (Armonk, NY: IBM Corp.). Calculations of prevalence for each risk factor were established. Descriptive data were presented as mean±SD or number (%). Comparisons between groups were carried out using independent-sample t-test, ANOVA, or chi-square tests, when appropriate. The associations between high hemoglobin and pregnancy complications in pregnant women were assessed by calculating the odds ratios (OR) and their 95% confidence intervals (CIs) using logistic regression. P-values of <0.05 were considered significant.

Institutional Review Board "IRB" at An-Najah National University (ANNU) in Palestine was fulfilled. Additional approval to get access to the medical records from the Palestinian Ministry of Health was obtained. All procedures followed were following the ethical standards of the responsible committee on human experimentation (institutional and national) and with the Helsinki Declaration of 1975, as revised in 2000. All data were collected and treated confidentially, kept safe, and available for the researchers only. Codes were used instead of names. Informed consent was waived as the study is a retrospective study and has no more than minimal risk. 

## Results

A total of (N=5263) medical records for pregnant women were collected, and 2698 records were excluded. The final number of eligible records was 2565. Table 1 describes the general characteristics of the pregnant women at registration and the 24th gestational week (24 GW). At registration, the mean values for maternal age were 26.9±5.8 years, ultrasound-based gestational age (8.18 ±2.34 weeks), Hb level (11.87 ±1.17 g/dl), FBS (79.7 ±8.60 mg/dl), systolic blood pressure (110.31 ±11.22 mmHg), and diastolic blood pressure (70.6 ±9.52 mmHg; Table 1). At registration, 32.4% of the women had high Hb levels, 47.0% had normal Hb levels, and 20.5% had low Hb levels. At 24 GW, 32.4% of the women had high Hb levels, 45.5% had normal Hb levels, and 22.1% had low Hb levels. The percentage of high FBS (≥126 mg/dl) at 24 GW was 4.4% and FBS (92 to <126 mg/dl) was 37.7% while 58.2% had normal FBS (<92 mg/dl). As previously mentioned, women who had FBS ≥92 mg/dl or systolic blood pressure ≥140 mmHg or diastolic blood pressure ≥90 mmHg either at registration or before that were excluded from this study.

**Table 1:** General characteristics of the pregnant women at registration and 24 gestational weeks Hb: hemoglobin, GW: gestational week, FBS: fasting blood sugar, mg/dl: milligram per deciliter, g/dl: gram per deciliter, mmHg: millimeter of mercury, SD: standard deviation, n: number

Character	Mean ± SD	Minimum–maximum
Maternal age at registration in years	26.9 ± 5.8	15–48
Gestational age at registration in weeks	8.18 ± 2.34	2–13
Gravidity (n)	2.61 ±1.64	1–17
Hb at registration (g/dl)	11.87 ± 1.17	7.3–16.5
Hb at 24 GW (g/dl)	11.84 ± 1.24	6.5–16.8
FBS at registration (mg/dl)	79.7 ± 8.60	50–91
FBS at 24 GW (mg/dl)	90.63 ±18.50	46–203
Systolic blood pressure at registration (mmHg)	110.31 ± 11.22	75–139
Systolic blood pressure at 24 GW (mmHg)	115.0 ± 13.03	79–179
Diastolic blood pressure at registration (mmHg)	70.6 ± 9.52	43–98
Diastolic blood pressure at 24 GW (mmHg)	74.0 ± 11.05	45-117
At registration	n (%)	
High Hb level (≥12.5 g/dl)	832(32.4)	
Normal Hb level (11 to <12.5 g/dl)	1206(47.0)	
Low Hb level (<11.0 g/dl)	527(20.5)	
At 24^th ^gestational week		
Hb level (≥12.5 g/dl)	830(32.4)	
Hb level (11 to <12.5 g/dl)	1165(45.5)	
Hb level (<11.0 g/dl)	565(22.1)	
FBS (≥126) mg/dl)	114(4.4)	
FBS (92 to <126 mg/dl)	957(37.3)	
FBS (<92 mg/dl)	1494(58.2)	
Systolic blood pressure (≥140 mmHg)	66(2.6)	
Diastolic blood pressure (≥90 mmHg)	136(5.3)	

Table 2 describes the percentages of different maternal FBS and blood pressure (BP) categories at 24 GW based on their Hb levels at registration. The percentages of high FBS ≥126 mg/dl at 24 GW in women who had high Hb levels at registration was 7.9% and was significantly higher than the percentages among women who had normal (2.7%) or low Hb (2.8%) at registration. The percentages of the FBS category (92 to <126 mg/dl) in women who had high Hb at registration (40.6%) was also significantly higher than percentages in women who had normal (34.7%) and low Hb (38.0%; p-value < 0.001). The percentage of high systolic (3.5%) or diastolic blood pressure (7.1%) at 24 GW was also higher among those who had high Hb at registration than those who had normal or low Hb at registration (p-value <0.05). Most of the women who had high Hb levels at registration had normal or low levels of Hb at 24 GW; however, 45.5% of the women maintained high Hb levels at 24 GW.

**Table 2:** The prevalence of different FBS and blood pressure categories at 24 GW among pregnant women based on their Hb levels at registration Hb: hemoglobin, GW: gestational week, FBS: fasting blood sugar, mg/dl: milligram per deciliter, g/dl: Gram per deciliter, mmHg: millimeter of mercury, CI: confidence interval, n: number

Variables at 24 GW	Categories	Hemoglobin levels in mg/dl at registration			P-value
		High Hb	Normal Hb	Low Hb	
		≥12.5	11 to <12.5	<11.0	
		n (%)	n (%)	n (%)	
FBS (mg/dl)	≥126	66(7.9)	33(2.7)	15(2.8)	<0.001
	92 to <126	338(40.6)	419(34.7)	200(38.0)	
	<92 mg/dl	428(51.4)	754(62.5)	32(59.2)	
Blood pressure (mmHg)	Systolic ≥ 140	29(3.5)	31(2.6)	6(1.1)	0.027
	Systolic < 140	803(96.5)	1175(97.4)	521(98.9)	
	Diastolic ≥ 90	59(7.1)	52(4.3)	25(4.7)	0.018
	Diastolic < 90	773(92.9)	1154(95.7)	502(95.3)	
	Both systolic ≥140 and diastolic blood pressure ≥90	17(2.0)	5(0.3)	1(0.2)	<0.001
	Either systolic ≥140 or diastolic blood pressure ≥90	54(6.5)	73(6.1))	29(5.6)	
	Neither systolic ≥140 nor diastolic blood pressure ≥90	761(91.5)	1128(93.5)	497(94.3)	
Hb (g/dl)	Hb ≥12.5	378(45.5)	353(29.3)	99(18.8)	<0.001
	Hb 11.0 to <12.5	350(42.1)	614(51.0)	201(38.2)	
	Hb <11.0	103(12.4)	236(19.6)	226(43.	

Table 3 describes the multinomial logistic regression for the association between adjusted Hb levels at registration and FBS levels at 24 GW. Compared to low Hb levels (<11.0 g/dl) at registration, high Hb ≥12.5 g/dl was significantly associated with high FBS (≥126 mg/dl) at 24 GW (OR=2.999, 95%CI: 1.675-5.368, p-value <0.001). Gravidity was significantly associated with decreased risk of FBS ≥126 mg/dl (OR=0.838, 95%CI: 0.704-0.991, p-value 0.039). No other significant associations were found between normal Hb levels (11-12.49 g/dl) at registration and FBS levels at 24 GW.

**Table 3:** Multi-nominal logistic regression model for the association between high Hb level at registration and increased fast blood sugar at 24 gestational weeks among pregnant women based on their hemoglobin levels at registration *Reference group is FBS <92 (mg/dl) Hb: hemoglobin, GW: gestational week, FBS: fasting blood sugar, mg/dl: milligram per deciliter, g/dl: Gram per deciliter, mmHg: millimeter of mercury, CI: confidence interval, n: number

FBS at 24 GW	Variables at registration	Cutoff	Odds ratio	95% (CI)	P-value
FBS ≥126 (mg/dl)*	Hb level g/dl	≥12.5	2.999	1.675–5.368	<0.001
		11 to <12.5	0.822	0.437–1.545	0.542
		Reference group <11.0			
	Maternal age in years		1.021	0.980–1.065	0.320
	Gravidity		0.838	0.704–0.991	0.039
	Ultrasound-based gestational age in weeks		0. 964	0.886–1.049	0.398
FBS 92 to <126 (mg/dl)*	Hb level g/dl	≥12.5	1.247	0.984–1.581	0.068
		11 to <12.5	0.878	0.702–1.098	0.254
		Reference group <11.0			
	Maternal age in years		1.011	0.993–1.0029	0.230
	Gravidity		0.940	0.882–1.003	0.062
	Ultrasound-based gestational age in weeks		0. 966	0.931–1.581	0.057

Table 4 describes the binary regression for the association between Hb levels at registration and systolic pressure at 24 GW. High Hb levels (≥12.5g/dl) at registration were significantly associated with increased risk of elevated systolic blood pressure ≥140 mmHg at 24 GW (OR=3.048, 95%CI: 1.252-7.42, p-value=0.014; Table 4).

**Table 4:** Model of binary logistic regression to assess the association between Hb levels at registration and systolic blood pressure at 24 GW *Reference group is at 24 GW <140 (mmHg) Hb: hemoglobin, GW: gestational week, FBS: fasting blood sugar, mg/dl: milligram per deciliter, g/dl: Gram per deciliter, mmHg: millimeter of mercury, CI: confidence interval, n: number

Systolic blood pressure at 24 GW ≥140 (mmHg)*	Cutoff	Odds ratio	95% (CI)	P-value
Hb level (g/dl) at registration	≥12.5	3.048	1.252–7.421	0.014
	11 to <12.5	2.318	0.959–5.598	0.062
	Reference group <11.0			
Maternal age (in years) at registration		0.991	0.941–1.043	0.732
Gravidity (n)		1.126	0.961–1.320	0.141
Ultrasound-based gestational age in weeks at registration (n)		1.034	0.930–1.149	0.539
FBS (mg/dl) at 24 GW	≥126	1.407	0.484–4.093	0.531
	92 to <126	1.287	0.771–2.149	0.334
	Reference group <92			

However, no association was found between high Hb at registration and increased risk of elevated diastolic blood pressure at 24 GW (p-value >0.05; Table 5).

**Table 5:** Model of binary logistic regression to assess the association between Hb levels at registration and diastolic blood pressure at 24 GW *Diastolic blood pressure at 24 GW <90 (mmHg) Hb: hemoglobin, GW: gestational week, FBS: fasting blood sugar, mg/dl: milligram per deciliter, g/dl: Gram per deciliter, mmHg: millimeter of mercury, CI: confidence interval, n: number

Diastolic blood pressure at 24 GW ≥90 (mmHg)*	Cutoff	Odds ratio	95% (CI)	P-value
Hb level (g/dl) at registration	≥12.5	1.440	0.877–2.362	0.149
	11 to <12.5	0.865	0.524–1.427	0.570
	Reference group <11.0			
Maternal age (in years) at registration		1.009	0.972–1.048	0.633
Gravidity (n)		0.975	0.850–1.117	0.714
Ultrasound-based gestational age in weeks at registration (n)		1.012	0.937–1.092	0.767
FBS (mg/dl) at 24 GW	≥126	1.571	0.780–3.165	0.206
	92 to <126	0.906	0.616–1.332	0.614
	Reference group <92			

## Discussion

Management of pregnancy complications includes identifying and early management of these complications and identifying high-risk patients. This study aimed to investigate the association between high Hb level at maternity care registration and various adverse health outcomes later in pregnancy (GDM and high blood pressure) among Palestinian women attending primary care centers. In agreement with previous studies, our results indicated that women who had high Hb (≥12.5 g/dl) at registration were at higher risk to have high FBS (≥126 mg/dl) at 24 GW (OR 3.39, p-value <0.001) [16]. This association suggests that having high Hb at registration in the first trimester increases the risk of developing GDM later in pregnancy. The reason for choosing 24 GW as a cutoff is because pregnant women at the primary care centers in Palestine get screened for GDM at that gestational age, which is consistent with the international recommendations [24]. These results could contribute to detecting high-risk pregnancies at registration in the first trimester among Palestinian women, therefore prompting more intensive GDM risk factors modification and closer follow-ups than those with normal Hb levels. Moreover, the biochemical basis of this association is probably due to the effect of iron on decreasing insulin sensitivity by altering the expression of insulin receptors in hepatocytes [25]. Further research exploring the impact of high Hb in the first trimester on GDM will help better understand the etiology and pathophysiology of GDM. This will ultimately lead to decreasing the consequences of GDM, including high birth weight, shoulder dystocia, birth injuries, neonatal hypoglycemia, and jaundice [26]. Moreover, pregnant women with high Hb in the first trimester are three times more likely (OR=3.048) to have an increased risk of having high systolic blood pressure (≥140 mmHg) at 24 GW (p-value=0.014) but not high diastolic blood pressure (p-value >0.05). This association between high Hb at registration in the first trimester and increased risk of gestational hypertension is consistent with a previous meta-analysis study [27]. However, that study confirms the association without specifying the type of HDP (systolic/diastolic/mixed) [27]. Hypertensive disorders in pregnancy remain the leading cause of maternal mortality worldwide [5]. Previous studies showed that high Hb level during pregnancy results from hypovolemia or hemoconcentration, which is usually the result of PE or pregnancy-induced hypertension [10, 11]. An obvious mechanism for blood pressure increase with increased Hb levels would be a result of the increased blood viscosity. It has been reported that the elevation of hematocrit and Hb levels increases blood viscosity and that increased viscosity through an effect on blood pressure may partly worsen cardiovascular function [28]. Another utility of this association lies in the early detection of high gestational hypertension risk pregnancies, prompting closer follow-ups and more intensive interventions to decrease its consequences on pregnant women, including progression to PE [29]. Other adverse effects of gestational hypertension include increased risk of cesarian delivery, preterm delivery, and intrauterine growth restriction [30]. According to a meta-analysis study, high Hb in the first trimester was associated with an increased risk of developing PE later in pregnancy [27]. Due to the lack of urine analysis in the medical records used in this study, this association could not be investigated. Further prospective studies involving this association between high Hb at registration and the risk of PE among Palestinian pregnant women are recommended.

This study has some limitations. The medical records did not include weight, body mass index, oral glucose tolerance test, and urine-analysis results, so the risk of PE could not be assessed and suggesting a problem of substandard quality of care [6,7]. Moreover, no fetal complications were recorded. However, the results of this study have important clinical implications for screening pregnant women. Moreover, the medical records did not include ferritin or iron levels. In addition, this study was confined to maternal pregnancy complications, without investigating fetal outcomes. Hb measurement is routinely done across the pregnancy as it is a relatively inexpensive and widely available test [9]. High Hb levels to identify GDM and HDP in this population could be an attractive early screening tool, especially in developing countries.

## Conclusions

Women who have a high hemoglobin level in their first trimester are at a higher risk of developing GDM and hypertension. Our findings suggest that Hb level at registration could be utilized in predicting the risk of GDM and HDP among Palestinian women who never had a previous history of these conditions. This early detection of high-risk pregnancies could lead to more intensive follow-ups or interventions, ultimately leading to decreased incidence and the adverse consequences of these conditions on pregnant women. We recommend considering high Hb at registration among Palestinian women as a risk factor for having GDM and HDP later in pregnancy. Moreover, we recommend conducting further research investigating the difference in adverse pregnancy conditions prognosis (GDM and HDP) when considering high Hb at registration as a risk factor compared to currently considered risk factors. Furthermore, since Hb measurements are an inexpensive and widely available test, we recommend conducting further research for the association between high maternal Hb and other adverse outcomes and fetal complications among Palestinian women. Further research is warranted about the exact pathophysiology of high Hb-induced isolated systolic hypertension and diabetes in pregnancy.
